# Long durations from symptom onset to diagnosis and from diagnosis to treatment in obsessive-compulsive disorder: A retrospective self-report study

**DOI:** 10.1371/journal.pone.0261169

**Published:** 2021-12-13

**Authors:** Sina Ziegler, Klara Bednasch, Sabrina Baldofski, Christine Rummel-Kluge

**Affiliations:** Department of Psychiatry and Psychotherapy, Medical Faculty, University of Leipzig, Leipzig, Germany; Medical University of Vienna, AUSTRIA

## Abstract

**Background:**

Although obsessive-compulsive disorder (OCD) is one of the most common mental disorders, it takes up to 17 years for patients with OCD to receive adequate therapy. According to existing outdated literature, this study aimed to investigate the current duration between symptom onset and diagnosis and between diagnosis and the beginning of therapy separately.

**Methods:**

In a cross-sectional study, *N* = 100 patients diagnosed with OCD undergoing treatment in a psychiatric outpatient department were assessed, using self-report questionnaires on sociodemographic and clinical variables. Based on self-reported information, the durations between age at symptom onset and age at diagnosis, and between age at diagnosis and beginning of therapy were calculated. To investigate associated factors, two subgroups of patients, one with a short duration between symptom onset and diagnosis < 7 years, and another with a long duration between symptom onset and diagnosis ≥ 7 years, respectively, were compared.

**Results and conclusion:**

Patients reported first symptoms of OCD at a mean age of 18.72 years. The mean duration between age at symptom onset and age at diagnosis was 12.78 years and the mean duration between age at diagnosis and the beginning of therapy was 1.45 years. Subgroup comparison indicated that patients with a short duration between symptom onset and diagnosis were significantly younger than patients with a long duration. However, patients with a short duration between symptom onset and diagnosis were at an older age when they reported first symptoms of OCD. Further, they showed less severe symptoms of OCD, higher functioning levels, and less self-stigmatization than patients with a long duration. It can be concluded that the duration until patients with OCD are diagnosed correctly and receive adequate treatment is still very long. Therefore, the duration between symptom onset and diagnosis should be shortened. Further, the duration between diagnosis and the beginning of therapy could be a good additional approach to reduce the overall duration of untreated disorder.

## Introduction

Obsessive-compulsive disorder (OCD) has an international lifetime prevalence of 2-3% and is, therefore, one of the most prevalent mental disorders [1, 2]. With a 12-month prevalence of 3.6%, OCD is one of the four most common mental disorders in Germany [3]. Despite the high prevalence, studies show that it takes up to 17 years from the onset of the first symptoms of OCD to the start of adequate therapy [4]. This long duration indicates that symptoms of OCD are more likely to be chronic, meaning that symptoms have already become consolidated and are present constantly over a certain period when OCD is first diagnosed and treated as such [5]. According to [5], this leads to a poorer prognosis. However, some of the studies investigating the duration between the onset of first symptoms and the start of therapy are more than 10 years old. In recent years the awareness of mental disorders has increased both in the general population and among medical experts [6, 7]. Mental health has moved more into the focus of society, politics, and medicine. In particular, great importance is attached to the prevention and early detection of mental disorders. For this reason, many information campaigns have been launched and the promotion of mental health care has been initiated [8, 9]. It is, therefore, interesting to investigate how the duration between the onset of first symptoms of OCD and the start of an adequate therapy might have changed during the last years.

The duration of untreated disorder and which influence it might have on treatment response has been the subject of frequent studies [10]. However, the duration between diagnosis and the start of therapy has hardly been investigated. Even after a correct diagnosis, other obstacles can impede the start of therapy. Considerable reasons are, e.g., a long waiting period for a free therapy slot, reluctance about the therapy, or stigma and shame concerning symptoms of OCD [11, 12]. Thus, it is interesting to investigate the period until diagnosis and until the start of a therapy separately.

Several reasons for the long duration between symptom onset and diagnosis of OCD are discussed in the literature. One reason includes the rate of misdiagnosis. Often OCD is not recognized as such and patients receive a wrong diagnosis or even treatment [13, 14]. The first point of contact for patients with symptoms of OCD is often a general practitioner, but the diagnosis of OCD is usually given later by a psychiatrist [15].

Another possible factor contributing to the long duration between symptom onset and diagnosis is the stigmatization both by the patients themselves and their environment. OCD is often concealed for a long time by the patients due to shame and fear of being stigmatized, which can lead to social withdrawal and thus, to delayed help-seeking [16].

First symptoms of OCD often appear in late childhood or adolescence [4]. Earlier studies reported that patients with an early disorder onset sought professional help later than those with a late onset [15]. However, there are various methodological approaches on how to determine the onset of OCD [17]. The onset of disorder can either be defined as the moment when patients notice first symptoms [18]; when these symptoms cause distress [19]; or when all diagnostic criteria are being fulfilled [10]. These different definitions lead to heterogeneous results regarding variables such as age at disorder onset or duration of untreated disorder and thus, to limited comparability of studies [20].

Furthermore, many patients show some symptoms of OCD long before an actual OCD can be diagnosed, i. e., long before they fulfill all diagnostic criteria for OCD [21]. Especially during this time of first symptom onset, early intervention and prevention programs would be useful and could prevent the progression of OCD even before a full-syndrome OCD develops [22, 23]. A recent study by [17] showed that it takes on average seven years for subthreshold symptoms, which means symptoms of an OCD that did not cause distress, to convert into a diagnosable full-syndrome OCD. This result indicates that the mean duration between age at symptom onset (ASO) and the age where OCD could be diagnosed is 7 years.

Considering these gaps in the current literature, this study aimed to investigate the duration between symptom onset and diagnosis (duration ASO-AD) and the duration between diagnosis and beginning of a therapy (duration AD-therapy) in a sample of patients undergoing outpatient treatment for OCD. Further, differences between patients with a short duration ASO-AD and a long duration ASO-AD regarding various sociodemographic and clinical variables were analyzed. A well comparable study from 2013 [15], which was also conducted in Leipzig, indicated that female patients and patients with a later onset of OCD sought professional help earlier than male patients and patients with an early onset of OCD. Therefore, another aim of this study was to verify this information.

## Materials and methods

### Procedure

The study was conducted at the psychiatric outpatient department, Department of Psychiatry and Psychotherapy at the University of Leipzig Medical Center, Germany. Patients currently undergoing outpatient treatment for OCD were offered to participate in the study. Inclusion criteria were age ≥ 18 years, current diagnosis of OCD according to the ICD-10 (ICD-10 F42.-) [24], adequate German language skills, reading and vision skills. Written informed consent was obtained before participation. The study was approved by the Ethics Committee of the Medical Faculty of the University of Leipzig.

The aim was to survey as many patients as possible who were receiving frequent outpatient psychiatric treatment for their OCD at the University of Leipzig Medical Center, ideally the entire sample. In total, *N* = 113 patients were included in the study. Of these, *N* = 100 patients completed the questionnaire, which represents a participation rate of 88.5%. The remaining 13 patients did not provide data despite consent and several reminders.

Data were collected from February to May 2019. The survey was mainly conducted as a web-based survey. Patients who could not or did not want to use the web-based version were given a paper-and-pencil version.

### Measures

The survey comprised validated questionnaires as well as items developed by the authors themselves. The following validated questionnaires were used: Obsessive-Compulsive-Inventory-Revised, German Adaptation (OCI-R) [25, 26], Global Assessment of Functioning (GAF) [27, 28], and Universal Stigma Scale (USS) [29].

The OCI-R is a self-report questionnaire assessing the severity of obsessive-compulsive symptoms and is reliable and valid [30]. It consists of 18 items and is scored on a five-point Likert scale ranging from 0 = “not at all” to 4 = “very strong”. It covers the six main symptom areas of OCD on six subscales (washing, checking, ordering, hoarding, neutralizing, and compulsive thoughts; each ranging from 0-12) and a total sum score (ranging from 0-72). Higher scores indicate higher levels of obsessive-compulsive symptoms.

The GAF is a diagnostic screening instrument administered by an expert to rate the patient’s psychosocial functioning level, which showed good reliability [31]. In this study, the GAF was assessed by the patients treating psychiatrist. The GAF scale is divided into 10 functioning levels with each of these being graded in ten steps from 1-100%. For the assessment, a single total score is defined which indicates the patient’s overall functional level. Lower levels indicate a lower functioning level.

The USS is a self-report stigma questionnaire assessing stigmatizing attitudes, which showed strong internal consistency and factor structure [29]. It was originally developed for assessing the stigmatizing attitudes towards people with eating disorders and major depressive disorder but is universally useable and customizable to any conditions. For this study, it was adapted for patients with OCD. The USS contains 11 items, rated on a 5-point Likert scale from 1 = “strongly agree” to 5 = “strongly disagree” and the total sum score ranges from 11-55. For this survey, the German translation from [32] was used. For better interpretability, the USS was inversely recoded so that higher scores indicate higher stigmatizing attitudes.

Furthermore, the questionnaire contained items on sociodemographic information (age, gender, marital status, educational level, employment status, and presence of children) and on information search regarding OCD, assessing if the patients had informed themselves independently about OCD at symptom onset and which media they had used for information search. The latter questions were taken from a study of participants at the 2nd German Patient Congress on Depression for Patients and Relatives 2013, Leipzig, Germany [33].

Moreover, to determine which discipline diagnosed OCD, patients were asked whether the diagnosis was given by a psychiatrist, psychologist, general practitioner, or someone else. Finally, psychiatric comorbidities were assessed for each patient by their treating psychiatrist.

### Duration between age at symptom onset and age at diagnosis

In addition to sociodemographic and clinical variables, the survey included questions on the following areas which were answered by the patients: age at symptom onset (ASO), which was assessed by reporting the age at which first symptoms of OCD occurred, age at diagnosis (AD), and duration between diagnosis and the beginning of therapy. The authors’ clinical experience has shown that patients are more likely to remember the onset of first symptoms of OCD rather than when they began to cause distress. The emotional interpretation of OCD related symptoms additionally seems to be very fluctuating at the beginning of the disorder. Therefore, it was determined that the age at which the first symptoms of OCD appeared should be used as the reference point. Further, patients surveyed in this study had already spent some time in therapy, dealing with the triggers or precipitating events of their OCD, and in the authors’ experience, can often pinpoint the onset of initial symptoms relatively accurately. Additionally, it is common practice to measure the symptom onset retrospectively and self-reported [34].

To avoid irritation the corresponding items were formulated as precisely as possible. Furthermore, the patients could ask questions in case of any ambiguities. The following item was defined to assess the age at symptom onset: “At what age did you experience first symptoms of an obsessive-compulsive disorder? Please enter the corresponding age in years.” To assess the age at diagnosis, the following item was defined: “When did you receive the diagnosis of an obsessive-compulsive disorder? This refers to the time when an OCD was detected in you. Please enter a number and accordingly days/weeks/months/years.” With this information and the age at answering the questionnaire, the age at diagnosis was calculated. Since it seems easier for most patients to remember how long ago they were diagnosed with OCD than to remember, or work out for themselves, how old they were at that time. Finally, the beginning of therapy was assessed as follows: “How long did it take for you to receive treatment for the first time after you were diagnosed? By treatment, we mean, for example, the start of therapy or the prescription of medication to treat your obsessive-compulsive disorder. Please enter a number and days/weeks/months/years accordingly.” In this way, an attempt was made to ensure that therapy specifically to OCD was meant. From this information, the duration between ASO and AD (duration ASO-AD, in years) and between AD and the beginning of a therapy (duration AD-therapy, in years) was calculated.

To investigate associated factors of the duration ASO-AD exploratory, the total sample was divided into two subgroups: (1) a group of patients with a short duration ASO-AD < 7 years; and (2) a group of patients with a long duration ASO-AD ≥ 7 years. The definitions of short and long duration ASO-AD, respectively, were based on the results of [17].

### Statistical analysis

Descriptive statistics were conducted to describe the characteristics of the total sample. To examine group differences between the two subgroups of patients with a short and long duration ASO-AD, respectively, *χ*^2^ tests were used for categorical variables (gender, marital status, educational level, employment status, presence of children, diagnosing person, independent information search, media used for information search, and psychiatric comorbidities).

Group differences between short and long duration ASO-AD on continuous variables (age, ASO, AD, duration ASO-AD, duration AD-therapy, OCI-R total score and subscale scores, GAF, and USS) were analyzed exploratory using Mann-Whitney *U* tests, as all continuous variables were non-normally distributed as determined by the Shapiro-Wilks test (all *p* < .05). Statistical analyses were performed using IBM SPSS version 24.0. A two-tailed *α* = 0.05 was applied for statistical testing. The effect sizes *r* and Φ, respectively, were interpreted as small = 0.10, medium = 0.30, and large = 0.50 [35].

## Results

### Description of the total sample

The total sample consisted of *N* = 100 patients. They were on average 40.04 years old (*SD* = 13.74) ranging from 18 to 82 years, *n* = 57 (57.0%) were female. Furthermore, *n* = 54 (54.0%) were not single (i. e., married or in a partnership). All sociodemographic and clinical characteristics are displayed in Table 1. The patients reported first symptoms (ASO) at the mean age of 18.72 years (*SD* = 11.24) and received their diagnosis of OCD (AD) at the mean age of 31.71 years (*SD* = 12.57). The mean duration ASO-AD was 12.78 years (*SD* = 11.30; range 0 to 45 years). Further, the mean duration between age at diagnosis and the beginning of a therapy (duration AD-therapy) was 1.45 years (*SD* = 4.51). Patients were diagnosed with OCD at a mean of 8.33 years (*SD* = 7.75) before participating in the study. Most patients (*n* = 72; 72.0%) were diagnosed with OCD by a psychiatrist. The psychosocial functioning level (GAF) was on average 68.69 (*SD* = 12.51), which corresponds to mild social impairments [36].

**Table 1:** Sociodemographic and clinical variables in the total sample.

Total sample (*N* = 100)		
	*M (SD)*	*n* (%)
**Age** (years)	40.04 (13.74)	
**Gender**		
Female		57 (57.0)
Male		42 (42.0)
Diverse		1 (1.0)
**Marital status**		
Not single		54 (54.0)
Single		46 (46.0)
**Educational level**		
Secondary education		15 (15.0)
Postsecondary non-tertiary education		15 (15.0)
Vocational education		34 (34.0)
Bachelor’s, Master’s, Doctoral or equivalent level		36 (36.0)
**Employment status**		
Employed		60 (60.0)
Unemployed		31 (31.0)
Unable to work		9 (9.0)
**Children**		
Yes		44 (44.0)
No		56 (56.0)
**Duration ASO-AD** (years)	12.78 (11.30)	
Age at symptom onset (ASO, years)	18.72 (11.24)	
Age at diagnosis (AD, years)	31.71 (12.57)	
**Duration AD-therapy** (years)	1.45 (4.51)	
**Duration AD-study participation** (years)	8.33 (7.45)	
**Diagnosing person**		
Psychiatrist		72 (72.0)
Psychotherapist		17 (17.0)
General practitioner		7 (7.0)
Others		4 (4.0)
**Severity of obsessive-compulsive symptoms** (OCI-R)		
Total score	24.53 (14.16)	
• Washing	3.85 (3.98)	
• Checking	5.31 (3.65)	
• Ordering	3.94 (3.70)	
• Hoarding	3.65 (2.78)	
• Neutralizing	2.60 (3.18)	
• Compulsive Thoughts	6.18 (3.57)	
**Functioning level** (GAF)	68.69 (12.51)	
**Self-stigmatization** (USS)	17.47 (5.80)	
**Independent information search**		
Yes		36 (36.0)
No		64 (64.0)
**Media used for information search**		
Digital media		30 (30.0)
Print media		20 (20.0)
TV/Radio		11 (11.0)
**Psychiatric comorbidities**		
None		46 (46.0)
One		35 (35.0)
Two or more		19 (19.0)

Duration ASO-AD: duration between age at symptom onset and age at diagnosis

Duration AD-therapy: duration between age at diagnosis and the beginning of a therapy

Duration AD-study participation: duration between age at diagnosis and the participation in the study

OCI-R: Obsessive-Compulsive-Inventory-Revised

GAF: Global Assessment of Functioning

USS: Universal Stigma Scale

Media used for information search: multiple answers were possible

### Group differences between patients with a short duration ASO-AD and a long duration ASO-AD

Concerning the exploratory variables, within the group with a short duration ASO-AD, the duration ASO-AD ranged from 0 to 5 years, whereas in the group with a long duration ASO-AD the duration ASO-AD ranged from 7 to 45 years. The two subgroups with a short duration and long duration ASO-AD, respectively, differed significantly in the following variables: age, ASO, AD, duration ASO-AD, OCI-R total score, OCI-R ordering and neutralizing subscales, functioning level (GAF), and self-stigmatization (USS); all *p* < .05; see Table 2. The effects were large for duration ASO-AD, medium for ASO, AD and age, and small for the remaining variables.

**Table 2:** Group differences between short and long duration ASO-AD.

	Short duration ASO-AD (*n* = 33)	Long duration ASO-AD (*n* = 62)	Test	Significance	Effect size
	*n* (%) or *M (SD)*	*n* (%) or *M (SD)*		*p*	
**Age** (years)	33.36 (11.86)	42.32 (13.23)	*U* = 589.00	.001	*r* = 0.35
**Gender**					
Female	21 (63.6)	33 (53.2)	*χ*^2^(2) = 3.21	.201	*ϕ* = 0.18
Male	11 (33.3)	29 (46.8)			
Diverse	1 (3.0)	0 (0.0)			
**Marital Status**					
Not single	20 (60.6)	32 (51.6)	*χ*^2^(1) = 0.70	.402	*ϕ* = 0.09
Single	13 (39.4)	30 (48.4)			
**Educational level**					
Secondary education	4 (12.1)	10 (16.1)	*χ*^2^(3) = 1.42	.702	*ϕ* = 0.12
Post-secondary non-tertiary education	4 (12.1)	10 (16.1)			
Vocational education	14 (42.4)	19 (30.6)			
Bachelor’s, Master’s, Doctoral or equivalent level	11 (33.3)	23 (37.1)			
**Employment status**					
Employed	23 (69.7)	37 (59.7)	*χ*^2^(2) = 2.28	.321	*ϕ* = 0.16
Unemployed	6 (18.2)	20 (32.3)			
Unable to work	4 (12.1)	5 (8.1)			
**Children**					
Yes	12 (36.4)	29 (46.8)	*χ*^2^(1) = 0.95	.329	*ϕ* = 0.10
No	21 (63.6)	33 (53.2)			
**Duration ASO-AD** (years)	1.85 (1.86)	18.60 (9.78)	*U* = 0.00	< .001	*r* = 0.82
Age at symptom onset (ASO; years)	22.85 (13.16)	16.08 (8.80)	*U* = 629.00	.002	*r* = 0.32
Age at diagnosis (AD; years)	24.70 (12.71)	34.68 (10.46)	*U* = 434.50	< .001	*r* = 0.45
**Duration AD-therapy** (years)	1.88 (5.62)	1.35 (4.08)	*U* = 861.00	.645	*r* = 0.05
**Duration AD-study participation** (years)	8.64 (7.91)	7.66 (7.57)	*U* = 968.50	.669	*r* = 0.04
**Diagnosing person**					
Psychiatrist	24 (72.7)	44 (71.0)	*χ*^2^(3) = 3.82	.282	*ϕ* = 0.20
Psychotherapist	4 (12.1)	13 (21.0)			
General practitioner	4 (12.1)	2 (3.2)			
Others	1 (3.0)	3 (4.8)			
**Severity of obsessive-compulsive symptoms** (OCI-R)					
**Total**	20.42 (13.00)	25.94 (13.83)	*U* = 753.00	.035	*r* = 0.22
• Washing	3.48 (4.20)	3.74 (3.72)	*U* = 920.00	.414	*r* = 0.08
• Checking	4.39 (3.02)	5.63 (3.73)	*U* = 837.50	.144	*r* = 0.15
• Ordering	2.82 (3.20)	4.50 (3.66)	*U* = 739.00	.025	*r* = 0.23
• Hoarding	2.03 (2.35)	2.92 (2.98)	*U* = 842.50	.151	*r* = 0.14
• Neutralizing	1.70 (2.47)	3.03 (3.41)	*U* = 744.00	.046	*r* = 0.20
• Compulsive thoughts	6.00 (3.38)	6.11 (3.64)	*U* = 1014.00	.944	*r* = 0.01
**Functioning level** (GAF)	73.67 (9.44)	66.90 (13.36)	*U* = 718.00	.016	*r* = 0.25
**Self-stigmatization** (USS)	14.94 (3.67)	18.71 (6.45)	*U* = 673.50	.006	*r* = 0.28
**Independent information search**					
Yes	15 (45.5)	18 (29.0)	*χ*^2^(1) = 2.56	.109	*ϕ* = 0.16
No	18 (54.5)	44 (71.0)			
**Media used for information search**					
Digital media	13 (39.4)	15 (24.2)	*χ*^2^(1) = 2.39	.122	*ϕ* = 0.16
Print media	7 (21.2)	11 (17.7)	*χ*^2^(1) = 0.17	.681	*ϕ* = 0.04
TV/Radio	2 (6.1)	8 (12.9)	*χ*^2^(1) = 1.07	.301	*ϕ* = 0.11
**Psychiatric comorbidities**					
None	18 (54.5)	26 (41.9)	*χ*^2^(2) = 3.37	.185	*ϕ* = 0.19
One	12 (36.4)	21 (33.9)			
Two or more	3 (9.1)	15 (24.2)			

Duration ASO-AD: duration between age at symptom onset and age at diagnosis

Short duration ASO-AD: duration < 7 years

Long duration ASO-AD: duration ≥ 7 years

Duration AD-therapy: duration between age at diagnosis and the beginning of a therapy

Duration AD-study participation: duration between age at diagnosis and the participation in the study

OCI-R: Obsessive-Compulsive-Inventory-Revised

GAF: Global Assessment of Functioning

USS: Universal Stigma Scale

Media used for information search: multiple answers were possible

Patients with a short duration ASO-AD were significantly younger, but reported their first symptoms (ASO) on average at an age of 22.85 years (*SD* = 13.16), whereas patients with a long duration ASO-AD reported their ASO at an age of 16.08 years (*SD* = 8.80).

Concerning the severity of symptoms of OCD, measured by the OCI-R, patients with a long duration ASO-AD showed in total significantly more severe symptoms of OCD than patients with a short duration ASO-AD, especially in the subcategories ordering and neutralizing. Furthermore, patients with a short duration ASO-AD displayed a significantly higher functioning level (GAF) and less self-stigmatization (USS) than patients with a long duration ASO-AD.

Groups showed no significant differences in gender, marital status, educational level, employment status, presence of children, duration AD-therapy, duration AD-study participation, diagnosing person, OCI-R subscales washing, checking, hoarding, and compulsive thoughts, independent information search, media used for information search, and the number of psychiatric comorbidities (small effects, all *p* > .05).

## Discussion

This study aimed to investigate the duration between symptom onset and diagnosis in patients with OCD and associated factors. Differences between two subgroups of patients with a short duration ASO-AD and a long duration ASO-AD were analyzed. As the main results of descriptive statistics, the mean duration ASO-AD was found to be 12.78 years and the mean duration AD-therapy was 1.45 years. Key results of subgroup comparison were that patients with a short duration ASO-AD were significantly younger, but reported first symptoms of OCD at an older age than patients with a long duration ASO-AD, which goes in line with our hypothesis. Contrary to our hypothesis, patients with a short duration ASO-AD were not more likely to be female. Further, patients with a short duration ASO-AD reported less severe symptoms of OCD, had a higher functioning-level, and showed less self-stigmatization than patients with a long duration ASO-AD.

### Discussion of the total sample

Considering the total sample, patients reported first symptoms of OCD at a mean age of 18.72 years. This is somewhat earlier than it was found in a comparable study, where first symptoms of OCD have been reported at a mean age of 23 years [15]. However, while discussing the results, it should be kept in mind that the data were collected retrospectively.

In older studies, the duration between symptom onset and the beginning of therapy was reported to be 17 years [4]. In this study, a mean duration ASO-AD of 12.78 years and a mean duration AD-therapy of 1.45 years was measured, indicating that the duration until the beginning of therapy is still quite long and shows only a slight reduction in the last years. A duration AD-therapy of 1.45 years further shows that patients, once they got the right diagnosis, start therapy with a rather long delay. Reasons could be a long waiting period for a free therapy slot, reluctance about the therapy, or lack of time due to employment. Other main reasons that were identified in a different study were stigma and shame concerning symptoms of OCD, worries about the ineffectiveness of therapy, and logistic and financial barriers [11]. This leads to further prolongation of the duration of untreated disorder and can contribute to the chronification of OCD. [37] reported a 31-day treatment delay in patients with bipolar disorder. This is a significantly shorter duration than the one measured in this study. However, hardly any other studies can be found that have separately investigated the duration between diagnosis and the beginning of therapy. Which makes it difficult to determine whether this long duration is specific to OCD.

Patients measured in this study reported rather low levels of self-stigmatization due to OCD. This result seems to be in contrast with other studies investigating the self-stigmatization of patients with mental disorders, e. g., depression or schizophrenia. These studies suggest that the self-stigmatization of patients with mental disorders is more likely to be high [38, 39] and that the expected self-stigmatization is the same in patients with OCD, depression, and schizophrenia [16]. Since these studies used different questionnaires and methods, the levels of stigmatization cannot be exactly compared. The USS, with which the level of self-stigmatization was measured in this study, was also used to measure stigmatizing attitudes towards eating disorders, obesity, and major depressive disorder [29]. In comparison, the level of self-stigmatization is the lowest in patients with OCD measured in this study. However, the scores measured in [29] were not assessed in a clinical sample but in undergraduate students who rated their stigmatizing attitudes towards people with different mental disorders, making the scores, not self-stigmatization scores.

The majority of patients were diagnosed with OCD by a psychiatrist and only 7.0% received their diagnosis by a general practitioner. It has to be considered if general practitioners might have difficulties in diagnosing OCD and if the awareness of OCD has to be increased [13, 14]. On the other hand, this could also indicate that general practitioners are well able to recognize when patients should be referred to a specialist. After all, psychiatrists have more experience and expertise in diagnosing and finally providing the right therapy, especially in patients with comorbidities.

### Discussion of the group differences

To investigate what distinguishes patients with a short duration ASO-AD from those with a long duration ASO-AD, the two groups were analyzed exploratory. Subgroup comparison showed that patients with a short duration ASO-AD were on average 33.36 years old and therefore significantly younger than patients with a long duration ASO-AD (*M* = 42.32 years). This is consistent with our expectation, as younger patients, due to their age, can be expected to have a shorter duration ASO-AD. However, it can be assumed that younger patients are more attached to digital media and therefore have better access to information concerning OCD in general and possible therapy options. Nevertheless, this cannot be confirmed by the data assessed. Moreover, patients with a short duration ASO-AD reported first symptoms at an older age than patients with a long duration ASO-AD, which goes in line with other studies [15].

Further, it is interesting to investigate the ranges of duration ASO-AD within the two subgroups. Patients with a short duration ASO-AD showed a very short mean duration ASO-AD of only 1.85 years, ranging from 0 to 5 years, whereas patients with a long duration ASO-AD reported a mean duration of 18.60 years ranging from 7 to 45 years. Thus, in this sample patients with OCD seem to be divided into those who search for professional help rather early and those who suffer from symptoms of OCD for a very long time before they receive the correct diagnosis. However, since the range within the subgroup long duration ASO-AD is very large, this could also be just a statistical issue. Furthermore, it is important to note that the data are exposed to a certain degree of uncertainty due to the retrospective design, as participants might in some cases not remember exactly when their symptoms started. Nevertheless, one possible reason could be the later symptom onset in the group with a short duration ASO-AD. Since their first symptoms appear at a young adult age (*M* = 22.85 years), they might be better able to seek professional help independently than minors. On the other hand, patients with a long duration ASO-AD reported first symptoms of OCD at a mean age of 16.08 years. This age in particular represents a vulnerable phase in the development of young people. Factors such as the incipient separation from the parental home on the one hand and the still existing dependence on the parents, on the other hand, make it difficult to seek professional help when psychiatric symptoms occur [40, 41]. It may be possible that during puberty, the pediatrician is reluctantly consulted due to shame and fear of being stigmatized and a connection to a general practitioner may often not yet established. In addition, the existing health insurance via the parents could raise the hurdle for seeking help.

Interestingly, there was no statistical difference between patients with a long and a short duration ASO-AD, respectively, regarding how many years before participating in the study the patients had been diagnosed (variable duration AD-study participation). This indicates that patients of both groups were on average diagnosed in the same year. In recent years, there has been a significant increase in psychiatric diagnoses, without an observed increase in overall prevalence [42]. This could be due to a growing awareness of mental health, which could make it easier for patients to seek professional help.

Concerning the severity of symptoms of OCD, patients with a short duration ASO-AD suffer from less severe symptoms than patients with a long duration ASO-AD. This could be an indication that OCD in patients with a short duration ASO-AD was not yet as chronified or that they have responded better to the therapy initiated. However, since therapy has already been initiated when patients participated in the study, only limited conclusions can be drawn. Interestingly, patients with a long duration ASO-AD reported significantly more severe symptoms in the subcategories ordering and neutralizing. Especially these two subcategories of OCD symptoms can be hidden from others for a long time and might not be as evident as other symptoms of OCD such as washing or controlling. A pronounced sense of order may generally be evaluated as positive and if it is judged by others to be excessive, it could be dismissed as eccentricity. Neutralizing is a compulsion that takes place mainly in thoughts and cannot be detected easily by other people. This might result in limited support in help-seeking by others. In comparison to the results of this study, [43] showed that patients with a chronic course of OCD reported more severe symptoms of OCD than patients with a non-chronic course. Further, they suffered more frequently from symmetry and ordering symptoms, and contamination and washing symptoms. This latter result seems to be partly opposite and partly similar to the result surveyed in this study since in this study patients with a long duration ASO-AD suffered from more severe symptoms in the subcategories ordering and neutralizing. [43] additionally hypothesize that the presence and severity of compulsions may be important in predicting chronic OCD.

The psychosocial functioning level was significantly higher in the subgroup with a short duration ASO-AD. As the cross-sectional study design does not allow for causal inferences, it might be possible that patients had a higher functioning level before they got their diagnosis and thus, were able to seek professional help independently or that they responded better to the therapy and therefore had an increase in psychosocial functioning.

Further, the self-stigmatization due to OCD was significantly lower in the group with a short duration ASO-AD. Presumably, a lower self-stigmatization could lead to an earlier help-seeking, which is supported by another study investigating the factors leading to a long duration of untreated disorder [44]. This study reported that believing that symptoms of OCD were not associated with an illness or can be overcome by oneself were important reasons for delayed treatment. Especially these two assumptions were part of the USS used in this study. However, since data were collected after the beginning of therapy, this group might also have responded better to therapeutic interventions such as psychoeducation or might have benefited from early intervention.

### Strength and limitations

The strengths of this study were the substantial sample size of 100 patients, the use of validated questionnaires, and expert ratings. Limitations include that the patients surveyed in this study were undergoing outpatient treatment at a university hospital and are, therefore, a special group of patients with, i. e. a higher level of education. This has the consequence that they might not be representative of the general population or patients who are severely impaired by OCD and receive inpatient therapy, for example. Further, data were collected cross-sectional and retrospective, meaning that patients had already started therapy and/or that therapy had been in progress for some time. Therefore, only limited conclusions can be drawn about the reasons for the differences in clinical variables in the two groups with a long and short duration ASO-AD, respectively. Another limitation concerns the reliability of self-reported information. Since the onset of OCD is sometimes far in the past, data on age at diagnosis are exposed to some uncertainties, such as inaccurate or distorted memories. Further, the beginning of clinical symptoms might not already justify a diagnosis of OCD, which could overestimate the duration of undiagnosed and untreated OCD. Finally, the split into the two compared subgroups leads to some limitations. Since the range of 7-38 years is very large for the group with a long duration ASO-AD, this may represent a heterogeneous group of patients. In addition, the division into two subgroups may limit the variance.

### Future work

The differences within the two groups short ASO-AD and long ASO-AD concerning the independent information search and which media was used for information search were not significant. However, it could be assumed that especially younger patients use digital media more frequently and therefore could benefit from digital support services. In the future it would be interesting to investigate the media usage behavior within patients who just got the diagnosis OCD and if the duration ASO-AD is shorter in this special group of patients. In addition, it would be interesting to explore reasons why patients do not start therapy immediately after receiving a diagnosis. A future paper will also investigate the influence of different comorbidities on the clinical course of OCD.

### Clinical implications

In summary, it can be concluded that the duration until patients with OCD receive adequate treatment is still very long. This is particularly relevant for patients with symptom onset in adolescence. To reduce this duration, very good cooperation and networking between psychiatrists in the pediatric and adult sector, and general practitioners, as well as pediatricians, would be beneficial. Especially the transition from child and adolescent mental health services to adult mental health services is a big challenge for both, patients and professionals [45, 46]. Since good integration into adult mental health services has an important influence on the further development of young adults, a formal transition system and collaborative planning are needed [46, 47]. In addition, awareness of OCD in general and possible symptoms should be increased in all professional disciplines.

Further, it is also important to shorten the duration between diagnosis and the start of treatment to enable patients to receive treatment early. In somatic medicine, for example, it would be unthinkable to leave a patient with diabetes mellitus untreated for another year before starting blood glucose control. It is important to inform patients correctly and in detail about their OCD and to advise them of the importance of prompt therapy. In addition, long waiting times for available therapy slots could be reduced by increasing professional staff and expanding therapy services.

Several additional factors could lead to a shortening of the duration ASO-AD and therefore a chronification of OCD could be prevented. It can be assumed that patients with a long duration ASO-AD, are more likely to have a chronic course of OCD. Since this leads to more severe symptoms and a higher disease burden [43], chronification should be prevented if possible. Especially early intervention has been discussed as a very important measure to prevent a severe progression of OCD [22]. Considerable factors that could lead to a shortening of the duration ASO-AD are easier access to information due to digital change and improved digital services for patients, which should be encouraged in the future. Further, raising awareness for OCD by general practitioners, psychiatrists, and population in general could contribute to this development, too.
